# Supplementary material for: Actin remodelling controls proteasome homeostasis upon stress
Source: Nat Cell Biol. 2022 Jun 23;24(7):1077–87. doi: 10.1038/s41556-022-00938-4 (PMC9276530; doi:10.1038/s41556-022-00938-4)
Supplement: Supplementary file 1 — Supplementary Table 1. [file 41556_2022_938_MOESM1_ESM.pdf]

nature portfolio

https://doi.org/10.1038/s41556-022-00938-4

Supplementary information

Actin remodelling controls proteasome homeostasis upon stress

In the format provided by the authors and unedited

Supplementary Table 1 | List of strains used in this study.

Strain	**Genotype**; plasmids in brakcets	Source
BY4741	MATa his3Δ1 leu2Δ0 met15Δ0 ura3Δ0	Horizon Discovery
BY4741 + FGH17	MATa his3Δ1 leu2Δ0 met15Δ0 ura3Δ0 [p416:FGH17::URA3]	This study
BY4741 + FGH17-5'UTRΔ	MATa his3Δ1 leu2Δ0 met15Δ0 ura3Δ0 [p416:FGH17-5'UTR Δ::URA3]	This study
BY4741 + FGH17-3'UTRΔ	MATa his3Δ1 leu2Δ0 met15Δ0 ura3Δ0 [p416:FGH17-3'UTR Δ::URA3]	This study
BY4741 + FGH17-70ntΔ	MATa his3Δ1 leu2Δ0 met15Δ0 ura3Δ0 [p416:FGH17-70nt Δ::URA3]	This study
BY4741 + FGH17-40ntΔ	MATa his3Δ1 leu2Δ0 met15Δ0 ura3Δ0 [p416:FGH17-40nt Δ::URA3]	This study
BY4741 + FGH17-23ntΔ	MATa his3Δ1 leu2Δ0 met15Δ0 ura3Δ0 [p416:FGH17-23nt Δ::URA3]	This study
rps6AΔ	MATa his3Δ1 met15Δ0 ura3Δ0 rps6a::LEU2	This study
rps18BΔ	MATa his3Δ1 met15Δ0 ura3Δ0 rps18b::LEU2	This study
ede1Δ	MATa his3Δ1 leu2Δ0 met15Δ0 ura3Δ0 ede1::kanMx	Horizon Discovery
cup1Δ	MATa his3Δ1 leu2Δ0 met15Δ0 ura3Δ0 cup1-1/2::kanMx	Horizon Discovery
Adc17-24xPP7SL + PCP-mKate2	MATa his3Δ1 leu2Δ0 met15Δ0 ura3Δ0 ADC17-24xPP7SL-LoxP [pFA6:cyc1p-PCP-mKate2::HIS3]	This study
Adc17-24xPP7SL Ede1-3xHA-GFPEnvy + PCP-mKate2	MATa his3Δ1 leu2Δ0 met15Δ0 ura3Δ0 ADC17-24xPP7SL-LoxP EDE1-3xHA-GFPENVY:KanMx [pFA6:cyc1p-PCP-mKate2::HIS3]	This study
Adc17-SunTag	MATa his3Δ1 leu2Δ0 met15Δ0 ura3Δ0 [p416:adc17p-Adc17-SunTag(24x)-PP7SL(24x)::URA3 + pFA6:cyc1p-PCP-EGFP(2X)-cyc1p-scFV-GCN4-mCherry::HIS3 +]	This study
Adc17-SunTag ede1Δ	MATa his3Δ1 leu2Δ0 met15Δ0 ura3Δ0 ede1::KanMx [p416:adc17p-Adc17-SunTag(24x)-PP7SL(24x)::URA3 + pFA6:cyc1p-PCP-EGFP(2X)-cyc1p-scFV-GCN4-mCherry::HIS3]	This study
Adc17-SunTag vrp1Δ	MATa his3Δ1 leu2Δ0 met15Δ0 ura3Δ0 vrp1::KanMx [p416:adc17p-Adc17-SunTag(24x)-PP7SL(24x)::URA3 + pFA6:cyc1p-PCP-EGFP(2X)-cyc1p-scFV-GCN4-mCherry::HIS3]	This study
Adc17-SunTag sla1Δ	MATa his3Δ1 leu2Δ0 met15Δ0 ura3Δ0 sla1::KanMx [p416:adc17p-Adc17-SunTag(24x)-PP7SL(24x)::URA3 + pFA6:cyc1p-PCP-EGFP(2X)-cyc1p-scFV-GCN4-mCherry::HIS3]	This study
syp1Δ	MATa his3Δ1 leu2Δ0 met15Δ0 ura3Δ0 syp1::kanMx	Horizon Discovery
clc1Δ	MATa his3Δ1 leu2Δ0 met15Δ0 ura3Δ0 clc1::kanMx	Horizon Discovery
chc1Δ	MATa his3Δ1 leu2Δ0 met15Δ0 ura3Δ0 chc1::kanMx	Horizon Discovery
pal1Δ	MATa his3Δ1 leu2Δ0 met15Δ0 ura3Δ0 pal1::kanMx	Horizon Discovery
yap1801Δ	MATa his3Δ1 leu2Δ0 met15Δ0 ura3Δ0 yap1801::kanMx	Horizon Discovery
yap1802Δ	MATa his3Δ1 leu2Δ0 met15Δ0 ura3Δ0 yap1802::kanMx	Horizon Discovery
alp1Δ	MATa his3Δ1 leu2Δ0 met15Δ0 ura3Δ0 alp1::kanMx	Horizon Discovery
alp3Δ	MATa his3Δ1 leu2Δ0 met15Δ0 ura3Δ0 alp3::kanMx	Horizon Discovery
aps2Δ	MATa his3Δ1 leu2Δ0 met15Δ0 ura3Δ0 aps2::kanMx	Horizon Discovery
apm4Δ	MATa his3Δ1 leu2Δ0 met15Δ0 ura3Δ0 apm4::kanMx	Horizon Discovery
ent1Δ	MATa his3Δ1 leu2Δ0 met15Δ0 ura3Δ0 ent1::kanMx	Horizon Discovery
ent2Δ	MATa his3Δ1 leu2Δ0 met15Δ0 ura3Δ0 ent2::kanMx	Horizon Discovery
end3Δ	MATa his3Δ1 leu2Δ0 met15Δ0 ura3Δ0 end3::kanMx	Horizon Discovery
sla1Δ	MATa his3Δ1 leu2Δ0 met15Δ0 ura3Δ0 sla1::kanMx	Horizon Discovery
lsb3Δ	MATa his3Δ1 leu2Δ0 met15Δ0 ura3Δ0 lsb3::kanMx	Horizon Discovery
lsb4Δ	MATa his3Δ1 leu2Δ0 met15Δ0 ura3Δ0 lsb4::kanMx	Horizon Discovery
lsb5Δ	MATa his3Δ1 leu2Δ0 met15Δ0 ura3Δ0 lsb5::kanMx	Horizon Discovery
ubx3Δ	MATa his3Δ1 leu2Δ0 met15Δ0 ura3Δ0 ubx3::kanMx	Horizon Discovery
gts1Δ	MATa his3Δ1 leu2Δ0 met15Δ0 ura3Δ0 gst1::kanMx	Horizon Discovery
ldb17Δ	MATa his3Δ1 leu2Δ0 met15Δ0 ura3Δ0 ldb17::kanMx	Horizon Discovery
bbc1Δ	MATa his3Δ1 leu2Δ0 met15Δ0 ura3Δ0 bbc1::kanMx	Horizon Discovery
aim21Δ	MATa his3Δ1 leu2Δ0 met15Δ0 ura3Δ0 aim21::kanMx	Horizon Discovery
ubp7Δ	MATa his3Δ1 leu2Δ0 met15Δ0 ura3Δ0 ubp7::kanMx	Horizon Discovery
bzz1Δ	MATa his3Δ1 leu2Δ0 met15Δ0 ura3Δ0 bzz1::kanMx	Horizon Discovery
vrp1Δ	MATa his3Δ1 leu2Δ0 met15Δ0 ura3Δ0 vrp1::kanMx	Horizon Discovery
myo3Δ	MATa his3Δ1 leu2Δ0 met15Δ0 ura3Δ0 myo3::kanMx	Horizon Discovery
myo5Δ	MATa his3Δ1 leu2Δ0 met15Δ0 ura3Δ0 myo5::kanMx	Horizon Discovery
rvs161Δ	MATa his3Δ1 leu2Δ0 met15Δ0 ura3Δ0 rvs161::kanMx	Horizon Discovery
rvs167Δ	MATa his3Δ1 leu2Δ0 met15Δ0 ura3Δ0 rvs167::kanMx	Horizon Discovery
vps1Δ	MATa his3Δ1 leu2Δ0 met15Δ0 ura3Δ0 vps1::kanMx	Horizon Discovery
Adc17-24xPP7SL + PCP-GFP(2x)	MATa his3Δ1 leu2Δ0 met15Δ0 ura3Δ0 ADC17-24xPP7SL-LoxP [pFA6:cyc1p-PCP-EGFP(2X)::HIS3]	This study
Adc17-24xPP7SL Abp140-3xHA-mKate2 + PCP-EGFP(2X)	MATa his3Δ1 leu2Δ0 met15Δ0 ura3Δ0 ADC17-24xPP7SL-LoxP ABP140-3xHA-mKATE2:KanMx [pFA6:cyc1p-PCP-EGFP(2X)::HIS3]	This study
Adc17-24xPP7SL Abp1-3xHA-mKate2 + PCP-EGFP(2X)	MATa his3Δ1 leu2Δ0 met15Δ0 ura3Δ0 ADC17-24xPP7SL-LoxP ABP1-3xHA-mKATE2:KanMx [pFA6:cyc1p-PCP-EGFP(2X)::HIS3]	This study
Adc17-24xPP7SL ede1Δ + PCP-EGFP(2X)	MATa his3Δ1 leu2Δ0 met15Δ0 ura3Δ0 ADC17-24xPP7SL-LoxP ede1Δ::LEU2 [pFA6:cyc1p-PCP-EGFP(2X)::HIS3]	This study
Adc17-24xPP7SL Ede1-aGFP + PCP-GFP(2x)	MATa his3Δ1 leu2Δ0 met15Δ0 ura3Δ0 ADC17-24xPP7SL-LoxP EDE1-aGFP:LEU2 [pFA6:cyc1p-PCP-EGFP(2X)::HIS3]	This study
ede1Δ + p416	MATa his3Δ1 leu2Δ0 met15Δ0 ura3Δ0 ede1::kanMx [p416]	This study
ede1Δ + p416-Ede1	MATa his3Δ1 leu2Δ0 met15Δ0 ura3Δ0 ede1::kanMx [p416-ede1p-Ede1]	This study
Adc17-24xPP7SL Ede1-tdimer2-aGFP + PCP-GFP(2x)	MATa his3Δ1 leu2Δ0 met15Δ0 ura3Δ0 ADC17-24xPP7SL-LoxP EDE1-aGFP:LEU2 [pFA6:cyc1p-PCP-EGFP(2X)::HIS3]	This study
Ede1-aGFP + p416-Adc17-24xPP7SL-Stop-24xMS2SL + PCP-GFP(2x) + MCP-mCherry	MATa his3Δ1 leu2Δ0 met15Δ0 ura3Δ0 EDE1-aGFP:LEU2 [p416-Adc17-24xPP7SL-Stop-24xMS2SL + pFA6:cyc1p-PCP-EGFP(2X)-cyc1p-MCP-mCherry::HIS3]	This study
adc17Δ + p416-Adc17-24xPP7SL-Stop-24xMS2SL + PCP-GFP(2x) + MCP-mCherry	MATa his3Δ1 leu2Δ0 met15Δ0 ura3Δ0 ede1::kanMx [p416-Adc17-24xPP7SL-Stop-24xMS2SL + pFA6:cyc1p-PCP-EGFP(2X)-cyc1p-MCP-mCherry::HIS3]	This study
adc17Δ Ede1-aGFP + p416-Adc17-24xPP7SL-Stop-24xMS2SL + PCP-GFP(2x) + MCP-mCherry	MATa his3Δ1 leu2Δ0 met15Δ0 ura3Δ0 ede1::kanMx EDE1-aGFP:LEU2 [p416-Adc17-24xPP7SL-Stop-24xMS2SL + pFA6:cyc1p-PCP-EGFP(2X)-cyc1p-MCP-mCherry::HIS3]	This study
Adc17-24xPP7SL Abp1-mKate2-aGFP + PCP-GFP(2x)	MATa his3Δ1 leu2Δ0 met15Δ0 ura3Δ0 ADC17-24xPP7SL-LoxP ABP1-mKate2-aGFP:LEU2 [pFA6:cyc1p-PCP-EGFP(2X)::HIS3]	This study
Adc17-24xPP7SL Abp1-mKate2 + PCP-GFP(2x)	MATa his3Δ1 leu2Δ0 met15Δ0 ura3Δ0 ADC17-24xPP7SL-LoxP ABP1-mKate2:KanMX [pFA6:cyc1p-PCP-EGFP(2X)::HIS3]	This study
Adc17-24xPP7SL Ede1-tdimer2 + PCP-GFP(2x)	MATa his3Δ1 leu2Δ0 met15Δ0 ura3Δ0 ADC17-24xPP7SL-LoxP EDE1-tdimer2:KanMX [pFA6:cyc1p-PCP-EGFP(2X)::HIS3]	This study
Adc17-24xPP7SL Sla1-mKate2 + PCP-GFP(2x)	MATa his3Δ1 leu2Δ0 met15Δ0 ura3Δ0 ADC17-24xPP7SL-LoxP SLA1-mKate2:KanMX [pFA6:cyc1p-PCP-EGFP(2X)::HIS3]	This study
Adc17-24xPP7SL Vrp1-mKate2 + PCP-GFP(2x)	MATa his3Δ1 leu2Δ0 met15Δ0 ura3Δ0 ADC17-24xPP7SL-LoxP VRP1-mKate2:KanMX [pFA6:cyc1p-PCP-EGFP(2X)::HIS3]	This study
Adc17-24xPP7SL Abp1-mKate2-aGFP + PCP-GFP(2x) ede1Δ	MATa his3Δ1 leu2Δ0 met15Δ0 ura3Δ0 ADC17-24xPP7SL-LoxP ede1Δ::LEU2 ABP1-mKate2:KanMX [pFA6:cyc1p-PCP-EGFP(2X)::HIS3]	This study
Adc17-70ntΔ (CRISPR/CAS9)	MATa his3Δ1 leu2Δ0 met15Δ0 ura3Δ0 5'UTR-70ntΔ-ADC17	This study
WT + FGH17-70ntd + Kozak	MATa his3Δ1 leu2Δ0 met15Δ0 ura3Δ0 ADC17-24xPP7SL-LoxP ABP1-mKate2:KanMX [pFA6:cyc1p-PCP-EGFP(2X)::HIS3]	This study
WT + FGH17-70nt only	MATa his3Δ1 leu2Δ0 met15Δ0 ura3Δ0 ADC17-24xPP7SL-LoxP ABP1-mKate2:KanMX [pFA6:cyc1p-PCP-EGFP(2X)::HIS3]	This study
BY4741 + FGH17-70ntΔ + Kozak	MATa his3Δ1 leu2Δ0 met15Δ0 ura3Δ0 [p416:FGH17-70ntΔ + Kozak::URA3]	This study
BY4741 + FGH17-70nt only	MATa his3Δ1 leu2Δ0 met15Δ0 ura3Δ0 [p416:FGH17--70nt only::URA3]	This study
act1-101	MATa his3Δ1 leu2Δ0 met15Δ0 ura3Δ0 act1-101:kanMX	Euroscraf
Rpl10-GFP + FGH17	MATa leu2Δ0 met15Δ0 ura3Δ0 RPL10-GFP:His3MX6 [p416:FGH17::URA3]	This study
Rpl10-GFP + FGH17-70ntΔ	MATa leu2Δ0 met15Δ0 ura3Δ0 RPL10-GFP:His3MX6 [p416:FGH17-70ntΔ::URA3]	This study
