# Supplementary material for: Actin remodelling controls proteasome homeostasis upon stress
Source: Nat Cell Biol. 2022 Jun 23;24(7):1077–87. doi: 10.1038/s41556-022-00938-4 (PMC9276530; doi:10.1038/s41556-022-00938-4)
Supplement: Supplementary file 2 — Reporting Summary [file 41556_2022_938_MOESM2_ESM.pdf]

nature portfolio

Corresponding author(s): Adrien Rousseau

Last updated by author(s): 13/04/2022

Reporting Summary

Nature Portfolio wishes to improve the reproducibility of the work that we publish. This form provides structure for consistency and transparency in reporting. For further information on Nature Portfolio policies, see our Editorial Policies and the Editorial Policy Checklist.

Statistics

For all statistical analyses, confirm that the following items are present in the figure legend, table legend, main text, or Methods section.

n/a	Confirmed	
☐	☒	The exact sample size (*n*) for each experimental group/condition, given as a discrete number and unit of measurement
☐	☒	A statement on whether measurements were taken from distinct samples or whether the same sample was measured repeatedly
☐	☒	The statistical test(s) used AND whether they are one- or two-sided<br>*Only common tests should be described solely by name; describe more complex techniques in the Methods section.*
☒	☐	A description of all covariates tested
☒	☐	A description of any assumptions or corrections, such as tests of normality and adjustment for multiple comparisons
☐	☒	A full description of the statistical parameters including central tendency (e.g. means) or other basic estimates (e.g. regression coefficient) AND variation (e.g. standard deviation) or associated estimates of uncertainty (e.g. confidence intervals)
☐	☒	For null hypothesis testing, the test statistic (e.g. F, t, r) with confidence intervals, effect sizes, degrees of freedom and P value noted<br>*Give P values as exact values whenever suitable.*
☒	☐	For Bayesian analysis, information on the choice of priors and Markov chain Monte Carlo settings
☒	☐	For hierarchical and complex designs, identification of the appropriate level for tests and full reporting of outcomes
☒	☐	Estimates of effect sizes (e.g. Cohen's d, Pearson's r), indicating how they were calculated

Our web collection on statistics for biologists contains articles on many of the points above.

Software and code

Policy information about availability of computer code

Data collection	ZEN 2.3 SP1 FP3 version 14.0.21.201, Proteome Discoverer software v.2.2, Chemidoc Touch imaging system, Zeiss LSM 880 Airyscan microscope, CFX384 Real-Time PCR Detection system, and Orbitrap Fusion Lumos mass spectrometer.
Data analysis	Images were acquired with Zeiss LSM 880 Airy Scan confocal microscope. Images taken by Zeiss confocal microscope were analysed using ZEN 2.3 SP1 FP3 black software (version 14.0.21.201). For quantifications of confocal images and Western Blot, Fiji ImageJ 2.1.0/1.53c software was used. For quantification of colocalization of red (translating mRNA) and green (all mRNA) puncta in Suntag experiments, the ComDet v.0.5.1. plugin was used. Proteomic data acquired by the Orbitrap Fusion Lumos mass spectrometer were analysed with Proteome Discoverer software v.2.2 with Mascot search engine. Gene expression data collected by the CFX384 Real-Time PCR Detection system was analysed with Bio-Rad CFX Maestro 2.0 (Version 5.0.021.0616). All statistics were performed using Graph Pad Prism 9 software (Version 9.1.2) (Graph Pad Software Inc., La Jolla, CA).

For manuscripts utilizing custom algorithms or software that are central to the research but not yet described in published literature, software must be made available to editors and reviewers. We strongly encourage code deposition in a community repository (e.g. GitHub). See the Nature Portfolio guidelines for submitting code & software for further information.

March 2021

Data

Policy information about availability of data

All manuscripts must include a data availability statement. This statement should provide the following information, where applicable:

- Accession codes, unique identifiers, or web links for publicly available datasets
- A description of any restrictions on data availability
- For clinical datasets or third party data, please ensure that the statement adheres to our policy

All the data generated or analysed during the current study are included in this published article and its supplementary files. The mass spectrometry proteomics data have been deposited to the ProteomeXchange Consortium via the PRIDE partner repository with the dataset identifier PXD027655.

Field-specific reporting

Please select the one below that is the best fit for your research. If you are not sure, read the appropriate sections before making your selection.

☒ Life sciences ☐ Behavioural & social sciences ☐ Ecological, evolutionary & environmental sciences

For a reference copy of the document with all sections, see nature.com/documents/nr-reporting-summary-flat.pdf

Life sciences study design

All studies must disclose on these points even when the disclosure is negative.

Sample size	No statistical test or power analysis were performed to predetermine sample size. We defined sample sizes based on routine practice in the similar studying fields. Experiments were repeated three or more time, as indicated.
Data exclusions	No data were excluded from the manuscript.
Replication	All experiments were replicated at least three time with similar findings. Sample sizes are provided in each figure legend.
Randomization	All images were acquired randomly.
Blinding	No blinding was use for this study. Experiments and data analyses were performed by the same investigator.

Reporting for specific materials, systems and methods

We require information from authors about some types of materials, experimental systems and methods used in many studies. Here, indicate whether each material, system or method listed is relevant to your study. If you are not sure if a list item applies to your research, read the appropriate section before selecting a response.

Materials & experimental systems

n/a	Involved in the study	
☐	☒	Antibodies
☐	☒	Eukaryotic cell lines
☒	☐	Palaeontology and archaeology
☒	☐	Animals and other organisms
☒	☐	Human research participants
☒	☐	Clinical data
☒	☐	Dual use research of concern

Methods

n/a	Involved in the study	
☒	☐	ChIP-seq
☒	☐	Flow cytometry
☒	☐	MRI-based neuroimaging

Antibodies

Antibodies used	Anti-Adc17 (Bertolotti laboratory; Rabbit; 1:1000), anti-Adc17-(2) (DSTT; Sheep; 1:250; DU66321; Fig. 7f,h), anti-Nas6 (Abcam; Rabbit; 1:2000; ab91447), anti-Flag (Sigma Aldrich; Mouse; 1:2000; F3165), anti-Rpt5 (Enzo life sciences; Rabbit; 1:5000; PW8245), anti-20S (Enzo life sciences; Rabbit; 1:2000; PW9355), anti-Mpk1 (Santa Cruz; Mouse; 1:500; sc-374434), anti-p-Mpk1 (Cell Signalling Technology; phospho-p44/42, Rabbit; 1:1000; 9101) and anti-p-Rps6 (Cell Signalling Technology; Rabbit; 1:1000; 2211). Anti-mouse IgG, HRP-linked Antibody (Cell Signalling Technology; 1:10000; #7076) and Anti-rabbit IgG, HRP-linked Antibody (Cell Signalling Technology; 1:10000; #7074).
Validation	Validation statement and product literature references are available here:<br><br>Rabbit and sheep anti-Adc17 antibodies, rabbit anti-Nas6 antibody, mouse anti-Mpk1 and rabbit anti-p-Mpk1antibodies have been validated using the respective knock-out yeast strains. Each antibody gave a specific signal at the expected size in WT cells while the

signal was absent in a strain where the target has been knocked-out. Re-expressing the target on a vector restored the signal, confirming the specificity of detection.

Mouse anti-Flag antibody is a gold standard extensively used in science (https://www.sigmaaldrich.com/GB/en/product/sigma/f3165?gclid=EAIaIQobChMIrtHQisap8gIVzNPtCh1yagiWEAAYASAAEgJopvD_BwE)

Rabbit anti-Rpt5 (https://www.enzolifesciences.com/BML-PW8245/proteasome-19s-rpt5-s6a-subunit-polyclonal-antibody/)

Rabbit anti-20S (https://www.enzolifesciences.com/BML-PW9355/proteasome-20s-core-subunits-polyclonal-antibody/)

Rabbit anti-Nas6 antibody (https://www.abcam.com/nas6-antibody-ab91447.html)

Mouse anti-Mpk1 antibody (https://www.scbt.com/fr/p/mpk1-antibody-d-1)

Mouse anti-p-Mpk1 antibody (https://www.cellsignal.com/products/primary-antibodies/phospho-p44-42-mapk-erk1-2-thr202-tyr204-antibody/9101) is extensively used in publications to monitor Mpk1 activation, including (Torres J et al., 2002; Liu L et al., 2018 and Sellers-Moya et al., 2021)

Rabbit anti-p-Rps6 antibody has been validated in yeast in these publications (Yerlikaya et al,. 2015 and Gonzalez A et al., 2015)

Anti-mouse IgG, HRP-linked Antibody (https://www.cellsignal.com/products/secondary-antibodies/anti-mouse-igg-hrp-linked-antibody/7076)

Anti-rabbit IgG, HRP-linked Antibody (https://www.cellsignal.com/products/secondary-antibodies/anti-rabbit-igg-hrp-linked-antibody/7074)

Eukaryotic cell lines

Policy information about cell lines

Cell line source(s)

BY4741 (MATa his3Δ1 leu2Δ0 met15Δ0 ura3Δ0) Horizon Discovery
BY4741 + FGH17 (MATa his3Δ1 leu2Δ0 met15Δ0 ura3Δ0 [p416:FGH17::URA3]) This study
BY4741 + FGH17-5'UTRΔ (MATa his3Δ1 leu2Δ0 met15Δ0 ura3Δ0 [p416:FGH17-5'UTRD::URA3]) This study
BY4741 + FGH17-3'UTRΔ (MATa his3Δ1 leu2Δ0 met15Δ0 ura3Δ0 [p416:FGH17-3'UTRD::URA3]) This study
BY4741 + FGH17-40ntΔ (MATa his3Δ1 leu2Δ0 met15Δ0 ura3Δ0 [p416:FGH17-40ntD::URA3]) This study
BY4741 + FGH17-30ntΔ (MATa his3Δ1 leu2Δ0 met15Δ0 ura3Δ0 [p416:FGH17-30ntD::URA3]) This study
BY4741 + FGH17-23ntΔ (MATa his3Δ1 leu2Δ0 met15Δ0 ura3Δ0 [p416:FGH17-23ntD::URA3]) This study
rps6AΔ (MATa his3Δ1 met15Δ0 ura3Δ0 rps6a::LEU2) This study
rps18BΔ (MATa his3Δ1 met15Δ0 ura3Δ0 rps18b::LEU2) This study
ede1Δ (MATa his3Δ1 leu2Δ0 met15Δ0 ura3Δ0 ede1::kanMx) Horizon Discovery
cup1-1/2Δ (MATa his3Δ1 leu2Δ0 met15Δ0 ura3Δ0 cup1-1/2::kanMx) Horizon Discovery
Adc17-24xPP7SL + PCP-mKate2 (MATa his3Δ1 leu2Δ0 met15Δ0 ura3Δ0 ADC17-24xPP7SL-LoxP [pFA6:cyc1p-PCP-mKate2::HIS3]) This study
Adc17-24xPP7SL Ede1-3xHA-GFPEnvy + PCP-mKate2 (MATa his3Δ1 leu2Δ0 met15Δ0 ura3Δ0 ADC17-24xPP7SL-LoxP EDE1-3xHA-GFPENVY:KanMx [pFA6:cyc1p-PCP-mKate2::HIS3]) This study
Adc17-SunTag (MATa his3Δ1 leu2Δ0 met15Δ0 ura3Δ0 [p416:adc17p-Adc17-SunTag(24x)-PP7SL(24x)::URA3 + pFA6:cyc1p-PCP-EGFP(2X)-cyc1p-scFV-GCN4-mCherry::HIS3]) This study
Adc17-SunTag ede1Δ (MATa his3Δ1 leu2Δ0 met15Δ0 ura3Δ0 ede1::KanMx [p416:adc17p-Adc17-SunTag(24x)-PP7SL(24x)::URA3 + pFA6:cyc1p-PCP-EGFP(2X)-cyc1p-scFV-GCN4-mCherry::HIS3]) This study
syp1Δ (MATa his3Δ1 leu2Δ0 met15Δ0 ura3Δ0 syp1::kanMx) Horizon Discovery
clc1Δ (MATa his3Δ1 leu2Δ0 met15Δ0 ura3Δ0 clc1::kanMx) Horizon Discovery
chc1Δ (MATa his3Δ1 leu2Δ0 met15Δ0 ura3Δ0 chc1::kanMx) Horizon Discovery
pal1Δ (MATa his3Δ1 leu2Δ0 met15Δ0 ura3Δ0 pal1::kanMx) Horizon Discovery
yap1801Δ (MATa his3Δ1 leu2Δ0 met15Δ0 ura3Δ0 yap1801::kanMx) Horizon Discovery
yap1802Δ (MATa his3Δ1 leu2Δ0 met15Δ0 ura3Δ0 yap1802::kanMx) Horizon Discovery
alp1Δ (MATa his3Δ1 leu2Δ0 met15Δ0 ura3Δ0 alp1::kanMx) Horizon Discovery
alp3Δ (MATa his3Δ1 leu2Δ0 met15Δ0 ura3Δ0 alp3::kanMx) Horizon Discovery
aps2Δ (MATa his3Δ1 leu2Δ0 met15Δ0 ura3Δ0 aps2::kanMx) Horizon Discovery
apm4Δ (MATa his3Δ1 leu2Δ0 met15Δ0 ura3Δ0 apm4::kanMx) Horizon Discovery
ent1Δ (MATa his3Δ1 leu2Δ0 met15Δ0 ura3Δ0 ent1::kanMx) Horizon Discovery
ent2Δ (MATa his3Δ1 leu2Δ0 met15Δ0 ura3Δ0 ent2::kanMx) Horizon Discovery
end3Δ (MATa his3Δ1 leu2Δ0 met15Δ0 ura3Δ0 end3::kanMx) Horizon Discovery
sla1Δ (MATa his3Δ1 leu2Δ0 met15Δ0 ura3Δ0 sla1::kanMx) Horizon Discovery
lsb3Δ (MATa his3Δ1 leu2Δ0 met15Δ0 ura3Δ0 lsb3::kanMx) Horizon Discovery
lsb4Δ (MATa his3Δ1 leu2Δ0 met15Δ0 ura3Δ0 lsb4::kanMx) Horizon Discovery
lsb5Δ (MATa his3Δ1 leu2Δ0 met15Δ0 ura3Δ0 lsb5::kanMx) Horizon Discovery
ubx3Δ (MATa his3Δ1 leu2Δ0 met15Δ0 ura3Δ0 ubx3::kanMx) Horizon Discovery
gts1Δ (MATa his3Δ1 leu2Δ0 met15Δ0 ura3Δ0 gst1::kanMx) Horizon Discovery
ldb17Δ (MATa his3Δ1 leu2Δ0 met15Δ0 ura3Δ0 ldb17::kanMx) Horizon Discovery
bbc1Δ (MATa his3Δ1 leu2Δ0 met15Δ0 ura3Δ0 bbc1::kanMx) Horizon Discovery
aim21Δ (MATa his3Δ1 leu2Δ0 met15Δ0 ura3Δ0 aim21::kanMx) Horizon Discovery
ubp7Δ (MATa his3Δ1 leu2Δ0 met15Δ0 ura3Δ0 ubp7::kanMx) Horizon Discovery
bzz1Δ (MATa his3Δ1 leu2Δ0 met15Δ0 ura3Δ0 bzz1::kanMx) Horizon Discovery
vrp1Δ (MATa his3Δ1 leu2Δ0 met15Δ0 ura3Δ0 vrp1::kanMx) Horizon Discovery

myo3Δ (MATa his3Δ1 leu2Δ0 met15Δ0 ura3Δ0 myo3::kanMx) Horizon Discovery
myo5Δ (MATa his3Δ1 leu2Δ0 met15Δ0 ura3Δ0 myo5::kanMx) Horizon Discovery
rvs161Δ (MATa his3Δ1 leu2Δ0 met15Δ0 ura3Δ0 rvs161::kanMx) Horizon Discovery
rvs167Δ (MATa his3Δ1 leu2Δ0 met15Δ0 ura3Δ0 rvs167::kanMx) Horizon Discovery
vps1Δ (MATa his3Δ1 leu2Δ0 met15Δ0 ura3Δ0 vps1::kanMx) Horizon Discovery
Adc17-24xPP7SL + PCP-GFP(2x) (MATa his3Δ1 leu2Δ0 met15Δ0 ura3Δ0 ADC17-24xPP7SL-LoxP [pFA6:cyc1p-PCP-EGFP(2X)::HIS3]) This study
Adc17-24xPP7SL Abp140-3xHA-mKate2 + PCP-EGFP(2X) (MATa his3Δ1 leu2Δ0 met15Δ0 ura3Δ0 ADC17-24xPP7SL-LoxP ABP140-3xHA-mKATE2:KanMx [pFA6:cyc1p-PCP-EGFP(2X)::HIS3]) This study
Adc17-24xPP7SL Abp1-3xHA-mKate2 + PCP-EGFP(2X) (MATa his3Δ1 leu2Δ0 met15Δ0 ura3Δ0 ADC17-24xPP7SL-LoxP ABP1-3xHA-mKATE2:KanMx [pFA6:cyc1p-PCP-EGFP(2X)::HIS3]) This study
Adc17-24xPP7SL ede1Δ + PCP-EGFP(2X) (MATa his3Δ1 leu2Δ0 met15Δ0 ura3Δ0 ADC17-24xPP7SL-LoxP ede1Δ::LEU2 [pFA6:cyc1p-PCP-EGFP(2X)::HIS3]) This study
Adc17-24xPP7SL Ede1-aGFP + PCP-GFP(2x) (MATa his3Δ1 leu2Δ0 met15Δ0 ura3Δ0 ADC17-24xPP7SL-LoxP EDE1-aGFP:LEU2 [pFA6:cyc1p-PCP-EGFP(2X)::HIS3]) This study
ede1Δ + p416 (MATa his3Δ1 leu2Δ0 met15Δ0 ura3Δ0 ede1::kanMx [p416]) This study
ede1Δ + p416-Ede1 (MATa his3Δ1 leu2Δ0 met15Δ0 ura3Δ0 ede1::kanMx [p416-ede1p-Ede1]) This study
Adc17-24xPP7SL Ede1-tdimer2-aGFP + PCP-GFP(2x) (MATa his3Δ1 leu2Δ0 met15Δ0 ura3Δ0 ADC17-24xPP7SL-LoxP EDE1-aGFP:LEU2 [pFA6:cyc1p-PCP-EGFP(2X)::HIS3]) This study
Ede1-aGFP + p416-Adc17-24xPP7SL-Stop-24xMS2SL + PCP-GFP(2x) + MCP-mCherry (MATa his3Δ1 leu2Δ0 met15Δ0 ura3Δ0 EDE1-aGFP:LEU2 [p416-Adc17-24xPP7SL-Stop-24xMS2SL + pFA6:cyc1p-PCP-EGFP(2X)-cyc1p-MCP-mCherry::HIS3]) This study
adc17Δ + p416-Adc17-24xPP7SL-Stop-24xMS2SL + PCP-GFP(2x) + MCP-mCherry (MATa his3Δ1 leu2Δ0 met15Δ0 ura3Δ0 ede1::kanMx [p416-Adc17-24xPP7SL-Stop-24xMS2SL + pFA6:cyc1p-PCP-EGFP(2X)-cyc1p-MCP-mCherry::HIS3]) This study
adc17Δ Ede1-aGFP + p416-Adc17-24xPP7SL-Stop-24xMS2SL + PCP-GFP(2x) + MCP-mCherry (MATa his3Δ1 leu2Δ0 met15Δ0 ura3Δ0 ede1::kanMx EDE1-aGFP:LEU2 [p416-Adc17-24xPP7SL-Stop-24xMS2SL + pFA6:cyc1p-PCP-EGFP(2X)-cyc1p-MCP-mCherry::HIS3]) This study
Adc17-24xPP7SL Abp1-mKate2-aGFP + PCP-GFP(2x) (MATa his3Δ1 leu2Δ0 met15Δ0 ura3Δ0 ADC17-24xPP7SL-LoxP ABP1-mKate2-aGFP:LEU2 [pFA6:cyc1p-PCP-EGFP(2X)::HIS3]) This study
Adc17-24xPP7SL Abp1-mKate2 + PCP-GFP(2x) (MATa his3Δ1 leu2Δ0 met15Δ0 ura3Δ0 ADC17-24xPP7SL-LoxP ABP1-mKate2:KanMX [pFA6:cyc1p-PCP-EGFP(2X)::HIS3]) This study
Adc17-24xPP7SL Abp1-mKate2 + PCP-GFP(2x) (MATa his3Δ1 leu2Δ0 met15Δ0 ura3Δ0 ADC17-24xPP7SL-LoxP ABP1-mKate2:KanMX [pFA6:cyc1p-PCP-EGFP(2X)::HIS3]) This study
Adc17-24xPP7SL Ede1-tdimer2 + PCP-GFP(2x) (MATa his3Δ1 leu2Δ0 met15Δ0 ura3Δ0 ADC17-24xPP7SL-LoxP EDE1-tdimer2:KanMX [pFA6:cyc1p-PCP-EGFP(2X)::HIS3]) This study
Adc17-24xPP7SL Sla1-mKate2 + PCP-GFP(2x) (MATa his3Δ1 leu2Δ0 met15Δ0 ura3Δ0 ADC17-24xPP7SL-LoxP SLA1-mKate2:KanMX [pFA6:cyc1p-PCP-EGFP(2X)::HIS3]) This study
Adc17-24xPP7SL Vrp1-mKate2 + PCP-GFP(2x) (MATa his3Δ1 leu2Δ0 met15Δ0 ura3Δ0 ADC17-24xPP7SL-LoxP VRP1-mKate2:KanMX [pFA6:cyc1p-PCP-EGFP(2X)::HIS3]) This study
Adc17-24xPP7SL Abp1-mKate2-aGFP + PCP-GFP(2x) ede1Δ (MATa his3Δ1 leu2Δ0 met15Δ0 ura3Δ0 ADC17-24xPP7SL-LoxP ede1Δ::LEU2 ABP1-mKate2:KanMX [pFA6:cyc1p-PCP-EGFP(2X)::HIS3]) This study
Adc17-70ntΔ (CRISPR/CAS9) (MATa his3Δ1 leu2Δ0 met15Δ0 ura3Δ0 5'UTR-70ntΔ-ADC17) This study
WT + FGH17-70ntd + Kozak (MATa his3Δ1 leu2Δ0 met15Δ0 ura3Δ0 ADC17-24xPP7SL-LoxP ABP1-mKate2:KanMX [pFA6:cyc1p-PCP-EGFP(2X)::HIS3]) This study
WT + FGH17-70nt only (MATa his3Δ1 leu2Δ0 met15Δ0 ura3Δ0 ADC17-24xPP7SL-LoxP ABP1-mKate2:KanMX [pFA6:cyc1p-PCP-EGFP(2X)::HIS3]) This study
BY4741 + FGH17-70ntΔ + Kozak (MATa his3Δ1 leu2Δ0 met15Δ0 ura3Δ0 [p416:FGH17-70ntΔ + Kozak::URA3]) This study
BY4741 + FGH17-70nt only (MATa his3Δ1 leu2Δ0 met15Δ0 ura3Δ0 [p416:FGH17--70nt only::URA3]) This study
act1-101 (MATa his3Δ1 leu2Δ0 met15Δ0 ura3Δ0 act1-101:kanMX) Euroscarf
Rpl10-GFP + FGH17 (MATa leu2Δ0 met15Δ0 ura3Δ0 RPL10-GFP:His3MX6 [p416:FGH17::URA3]) This study
Rpl10-GFP + FGH17-70ntΔ (MATa leu2Δ0 met15Δ0 ura3Δ0 RPL10-GFP:His3MX6 [p416:FGH17-70ntΔ::URA3]) This study

Authentication

Authentication of cell lines that were not generated in this study, was done by PCR and on the basis of the expected phenotype.

Mycoplasma contamination

Testing for Mycoplasma contamination was not needed, as only yeast has been used in this study

Commonly misidentified lines
(See ICLAC register)

No commonly misidentified cell lines were used
