# Supplementary figures and images for: Actin remodelling controls proteasome homeostasis upon stress
Source: Nat Cell Biol. 2022 Jun 23;24(7):1077–87. doi: 10.1038/s41556-022-00938-4 (PMC9276530; doi:10.1038/s41556-022-00938-4)

Fig. 1a

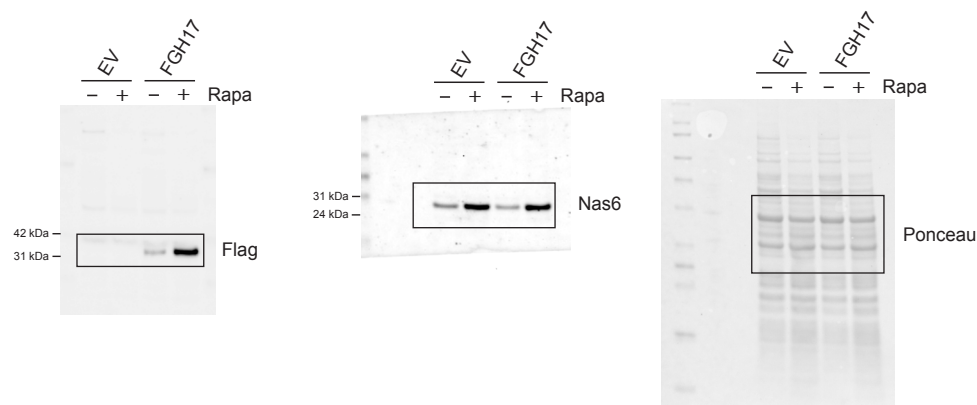

Fig. 1c

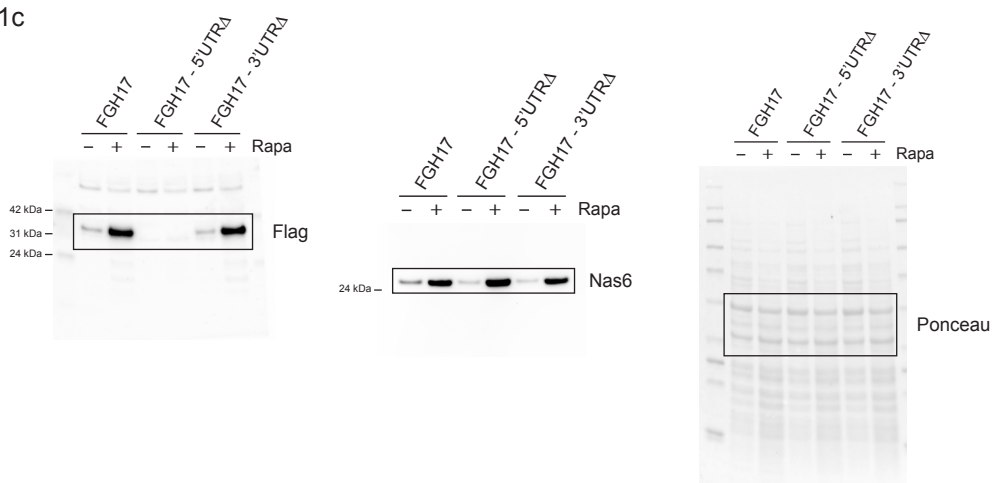

Fig. 1d

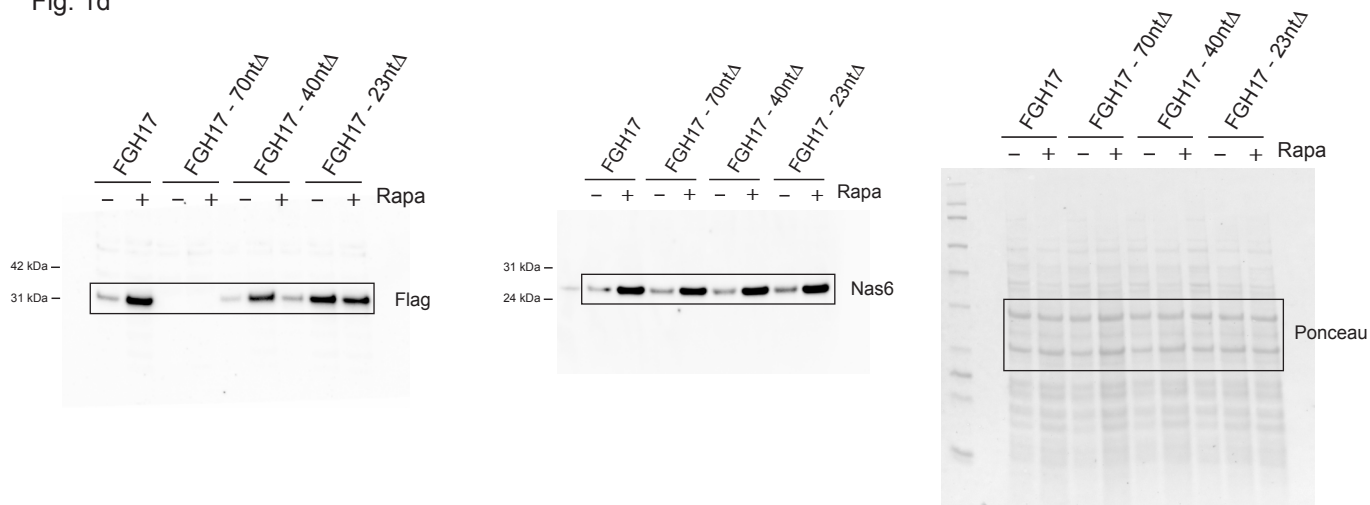

Fig. 1f

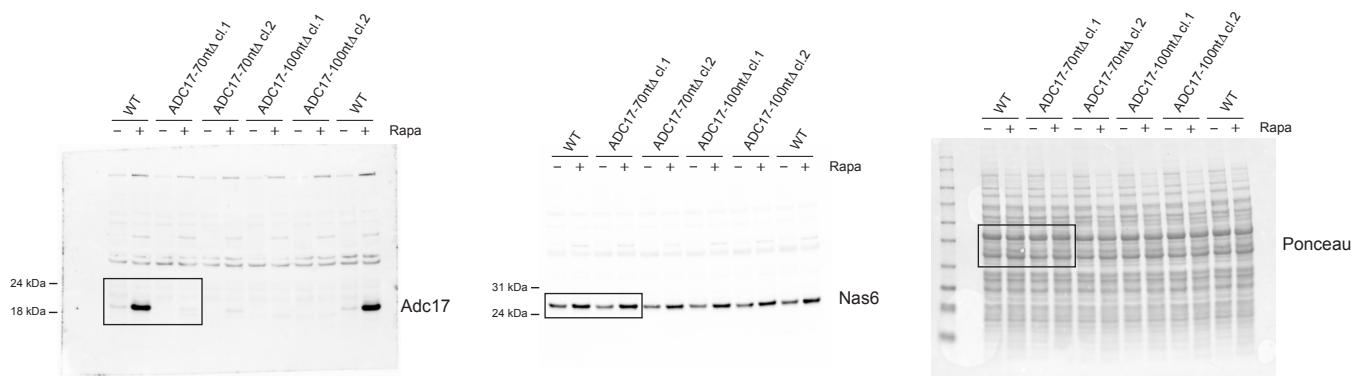

Supplement: Supplementary file 13 — Unprocessed western blots and/or gels. [file 41556_2022_938_MOESM13_ESM.pdf]

Fig. 2b

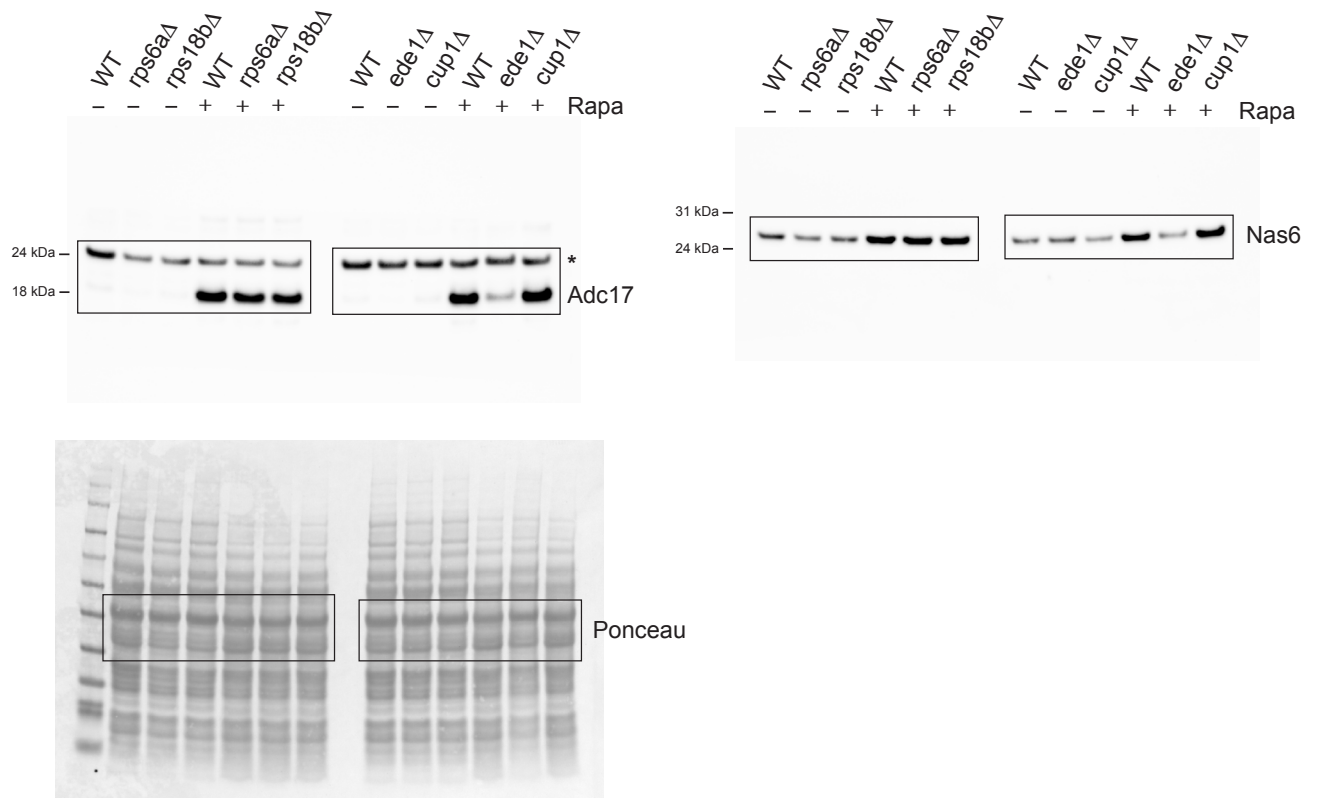

Fig. 2c

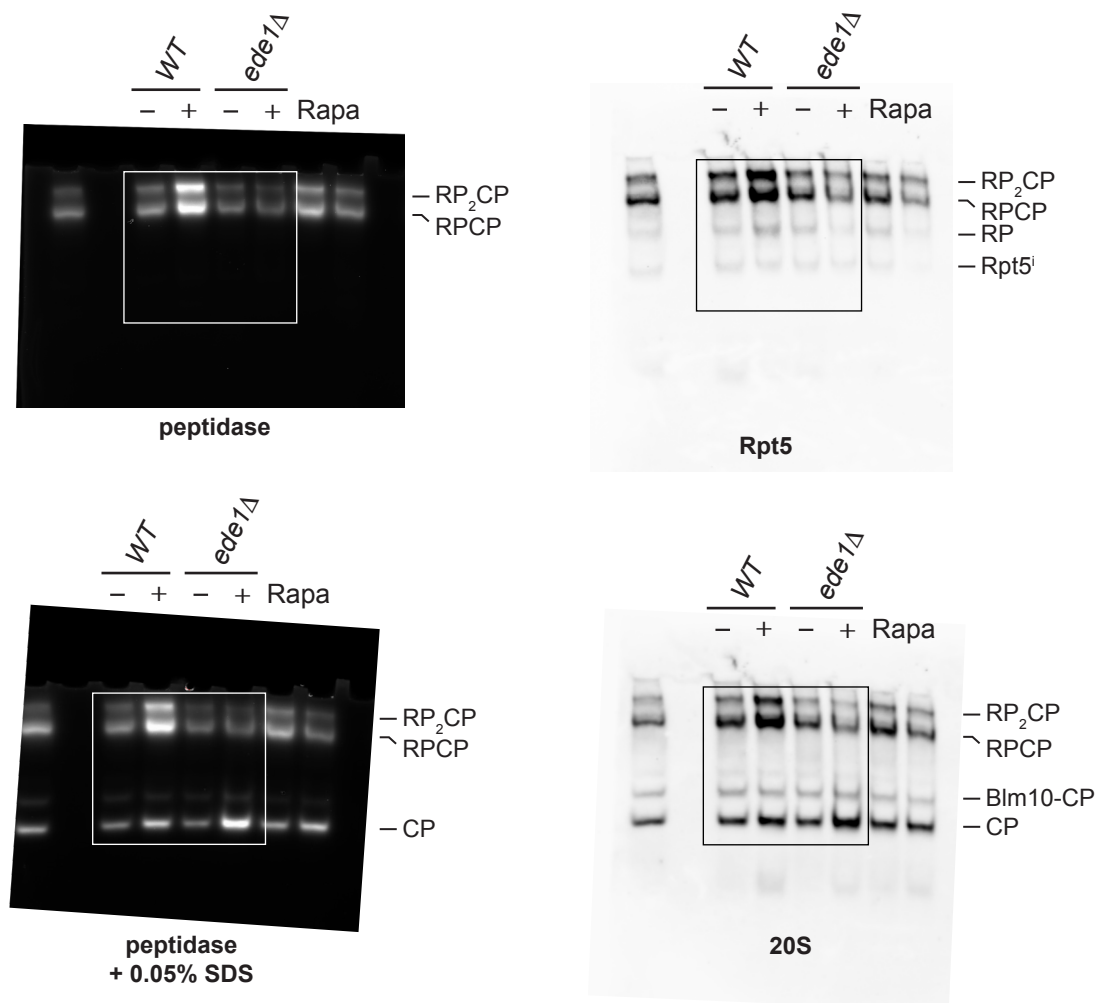

Supplement: Supplementary file 14 — Unprocessed western blots and/or gels. [file 41556_2022_938_MOESM14_ESM.pdf]

Fig. 4b

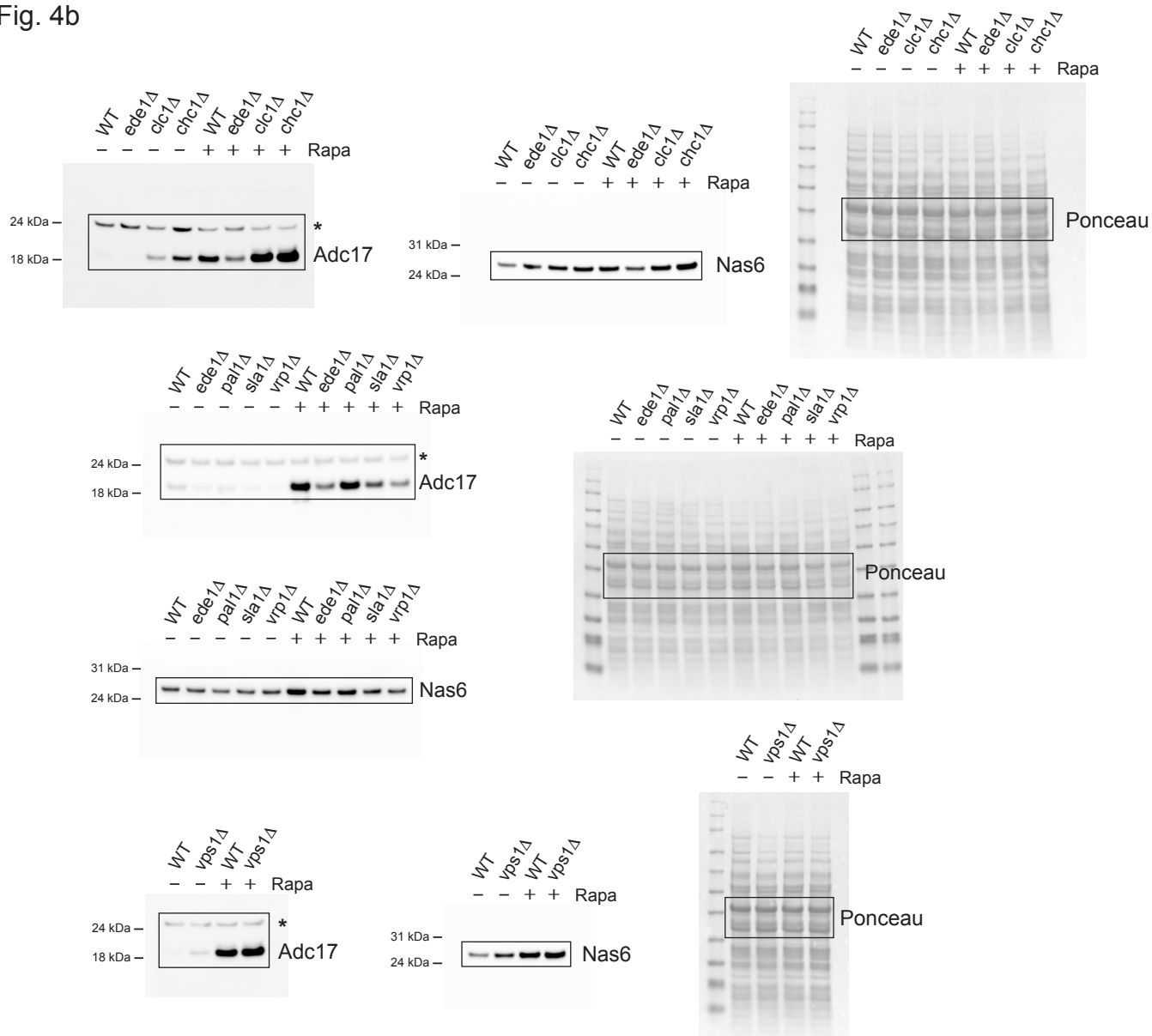

Fig. 4d

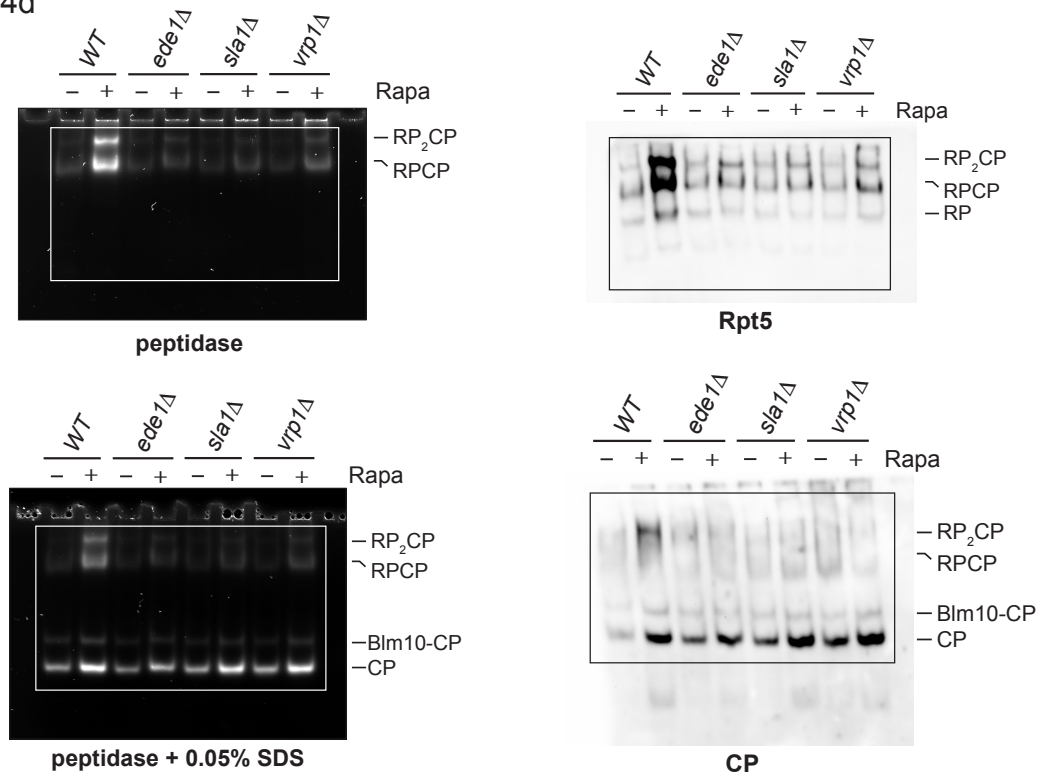

Supplement: Supplementary file 17 — Unprocessed western blots and/or gels. [file 41556_2022_938_MOESM17_ESM.pdf]

Fig. 5g

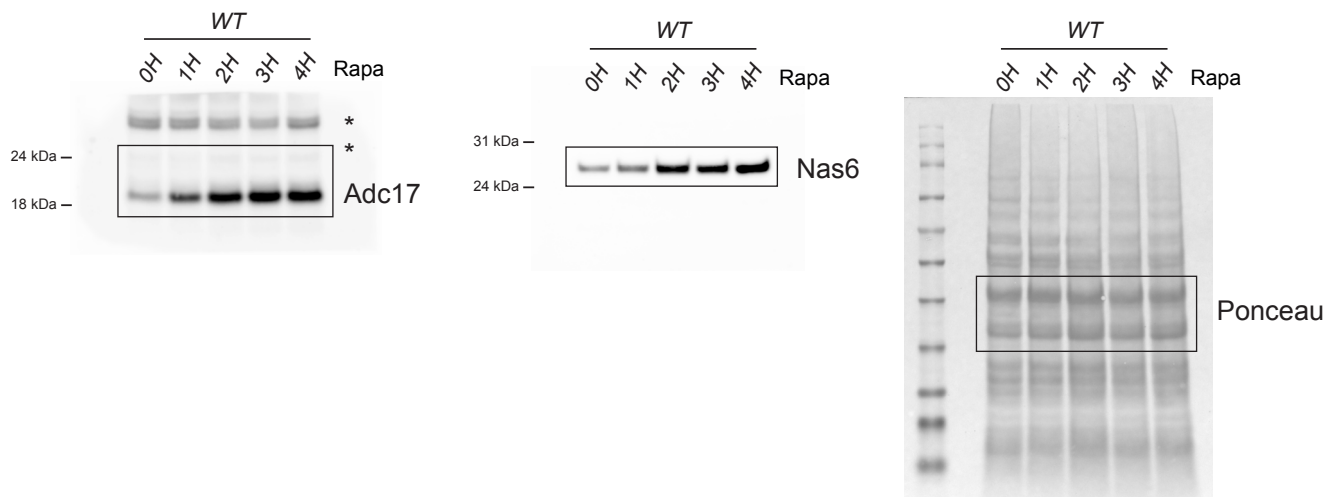

Supplement: Supplementary file 19 — Unprocessed western blots and/or gels. [file 41556_2022_938_MOESM19_ESM.pdf]

Fig. 6c

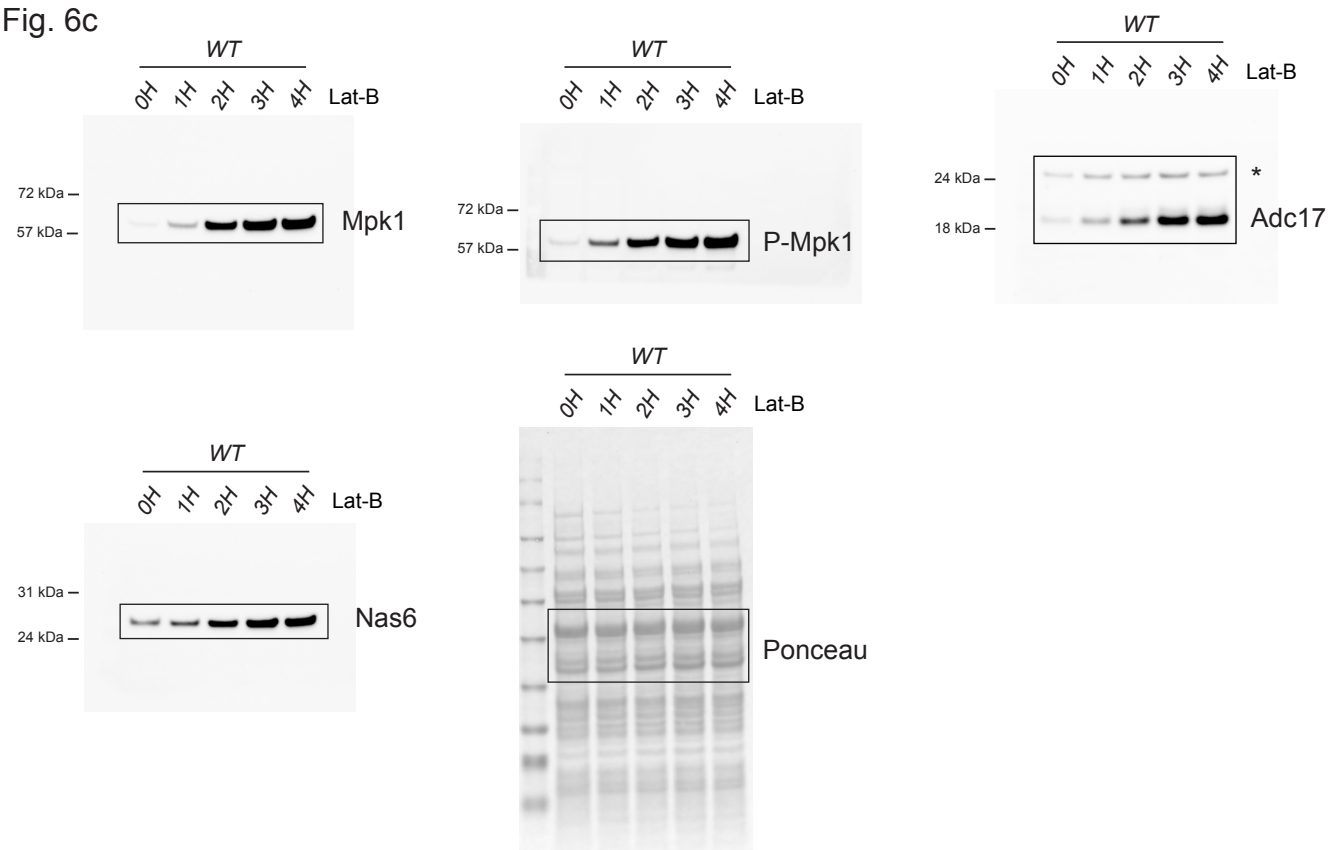

Fig. 6d

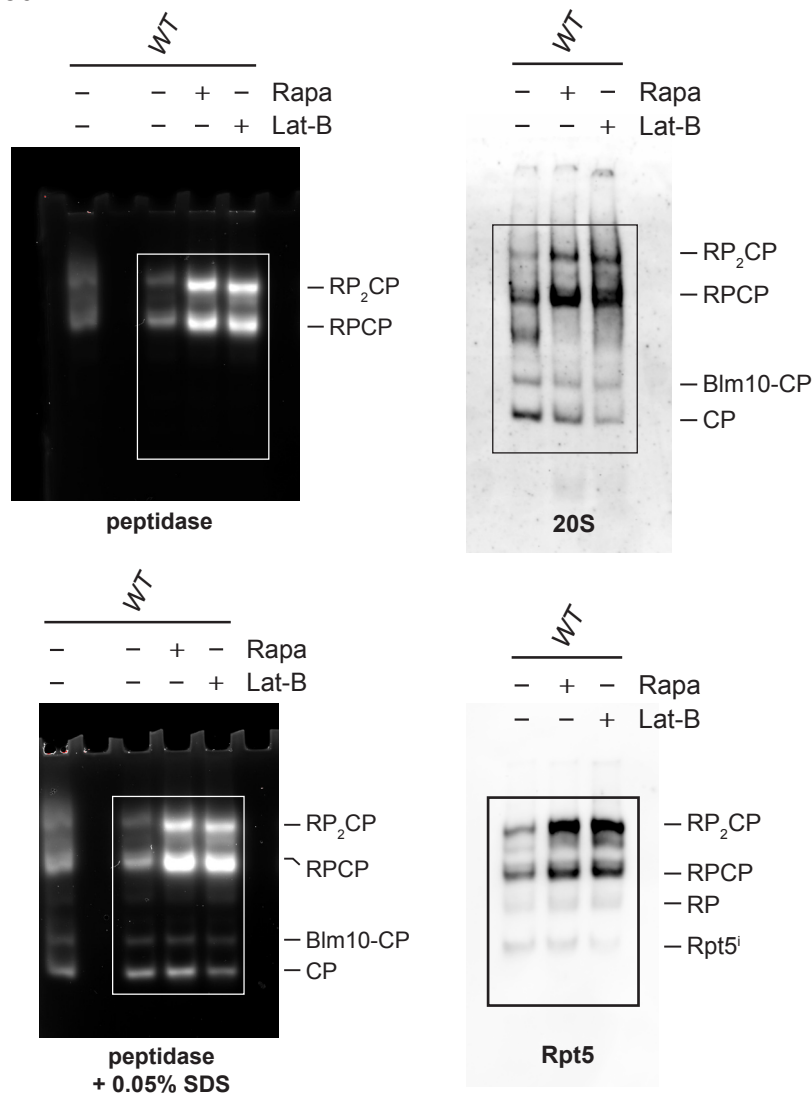

Supplement: Supplementary file 21 — Unprocessed western blots and/or gels. [file 41556_2022_938_MOESM21_ESM.pdf]

Fig. 7b

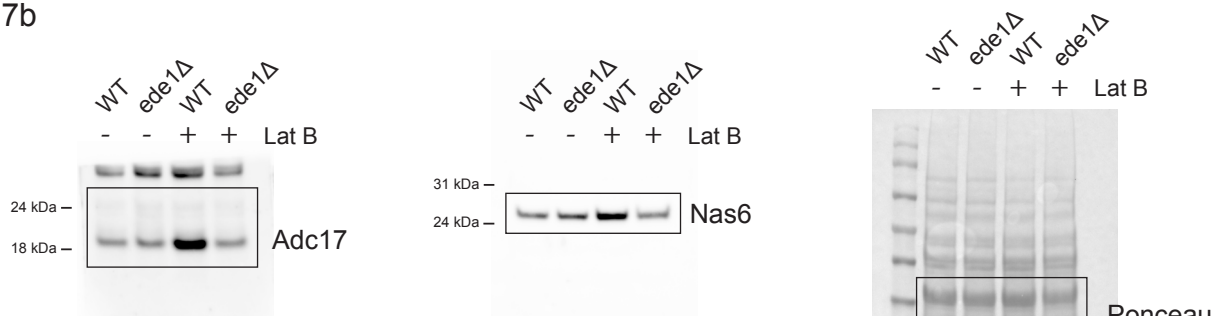

Fig. 7f

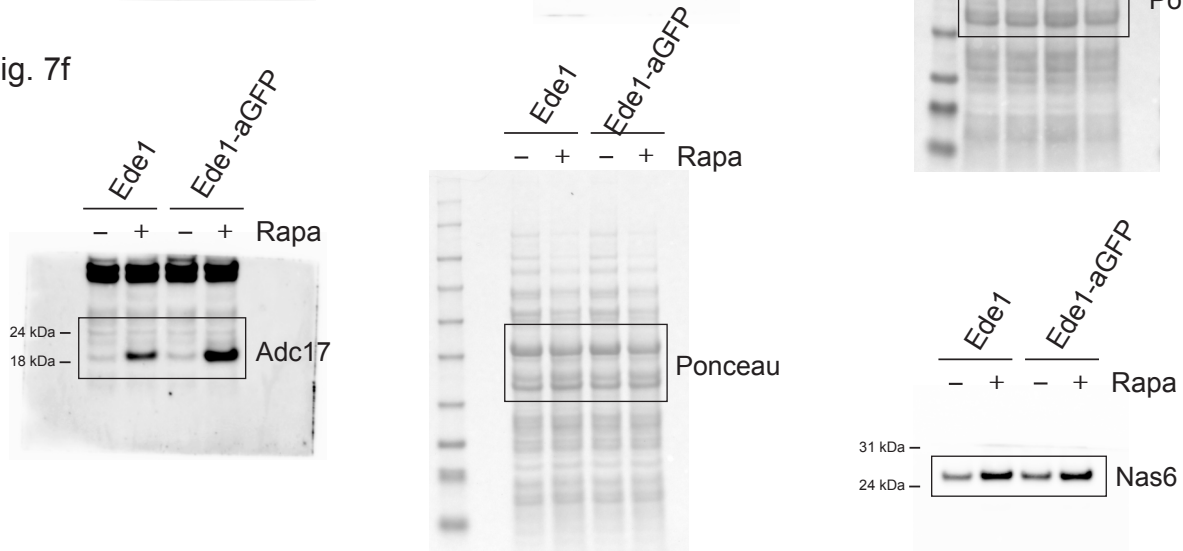

Fig. 7h

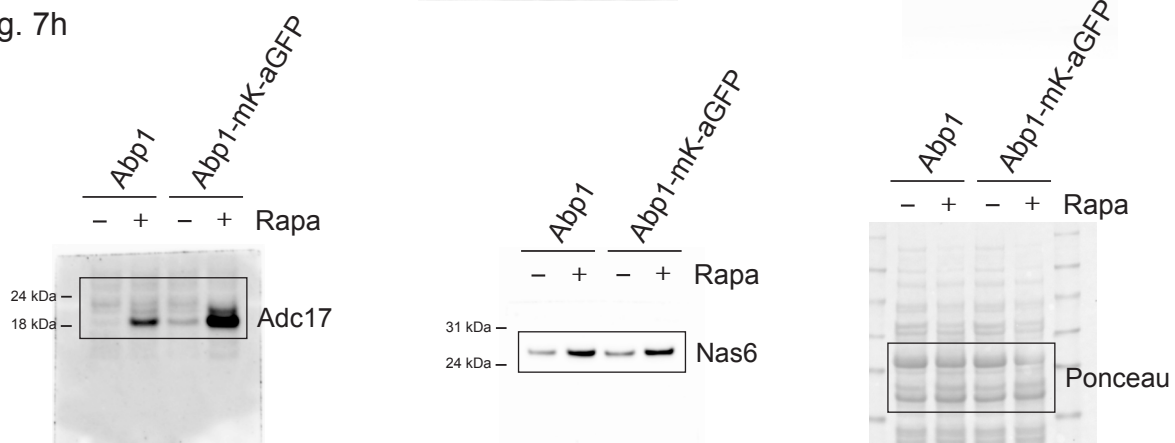

Fig. 7j

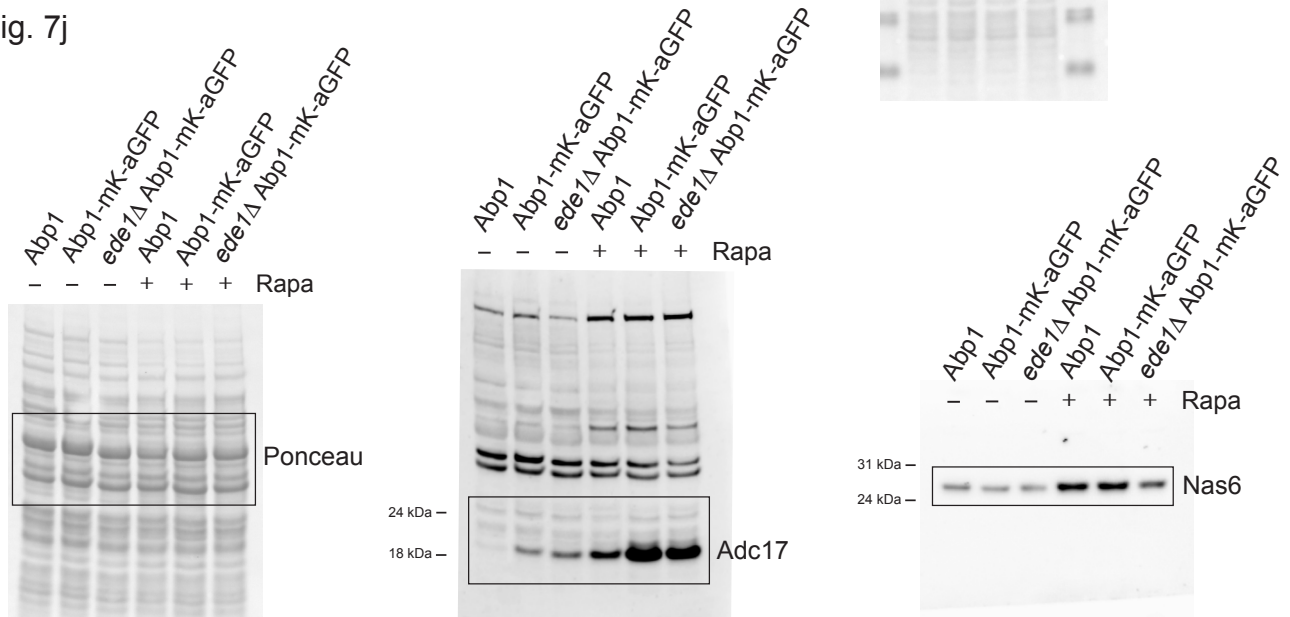

Supplement: Supplementary file 23 — Unprocessed western blots and/or gels. [file 41556_2022_938_MOESM23_ESM.pdf]

Extended Data Fig. 1a

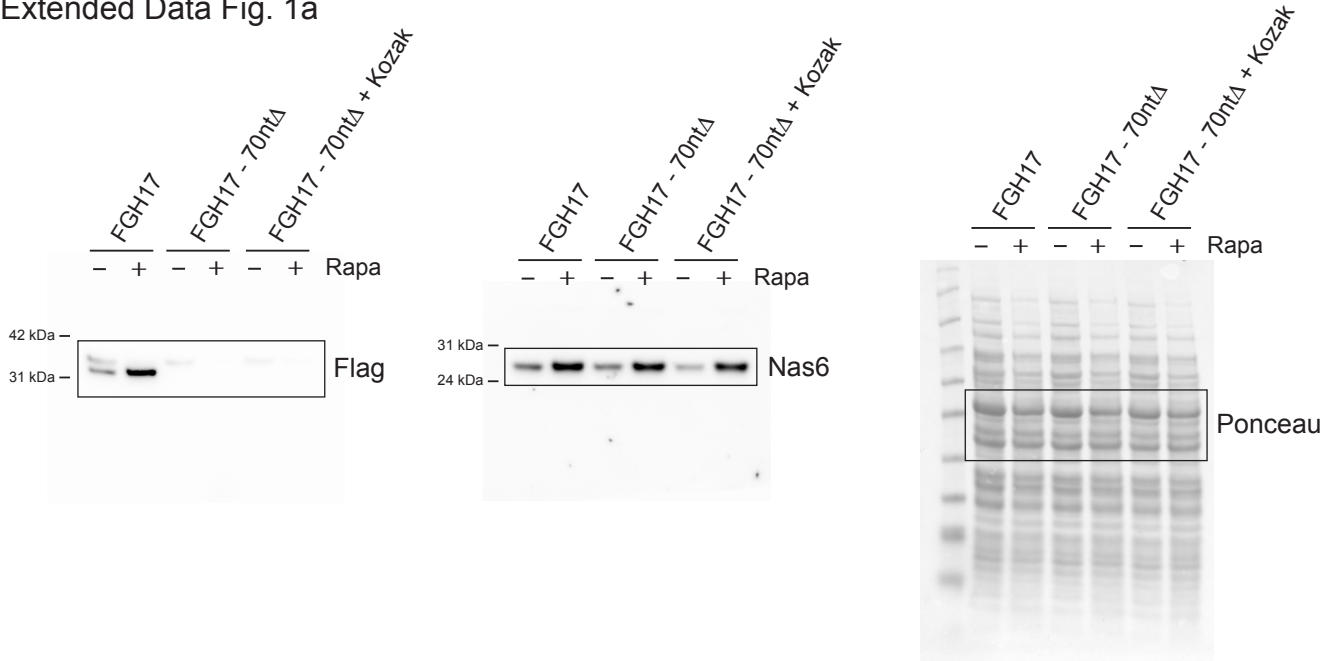

Extended Data Fig. 1b

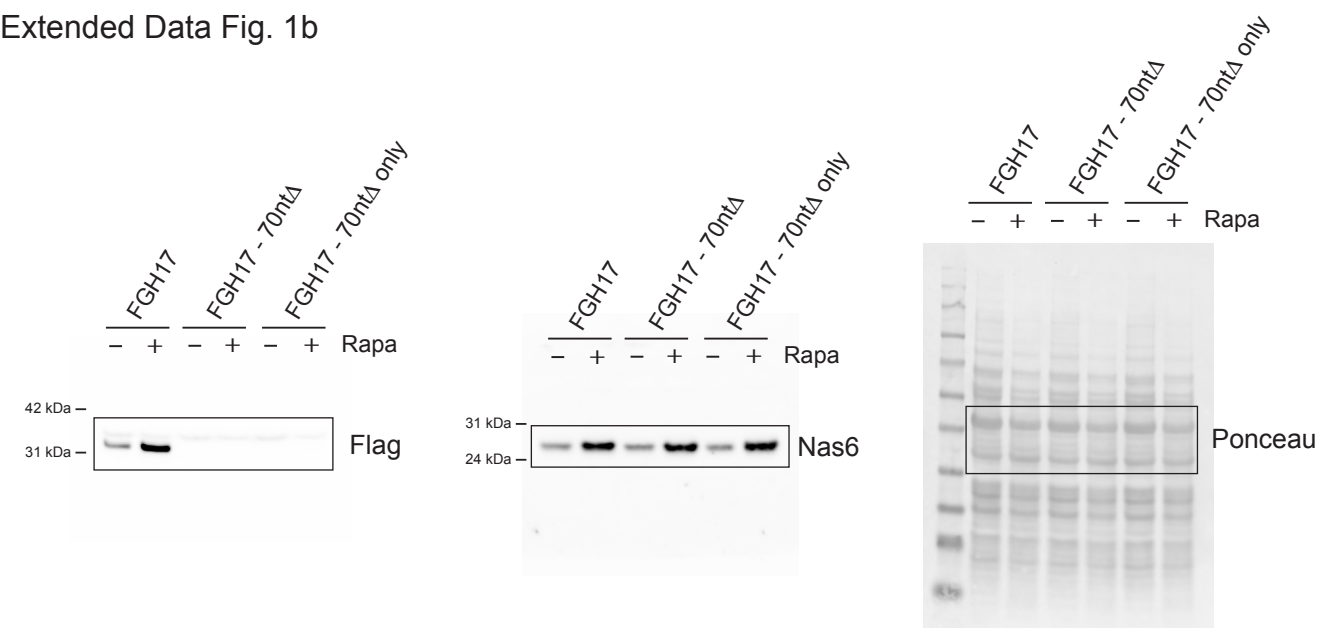

Supplement: Supplementary file 25 — Unprocessed western blots and/or gels. [file 41556_2022_938_MOESM25_ESM.pdf]

Extended Data Fig. 2b

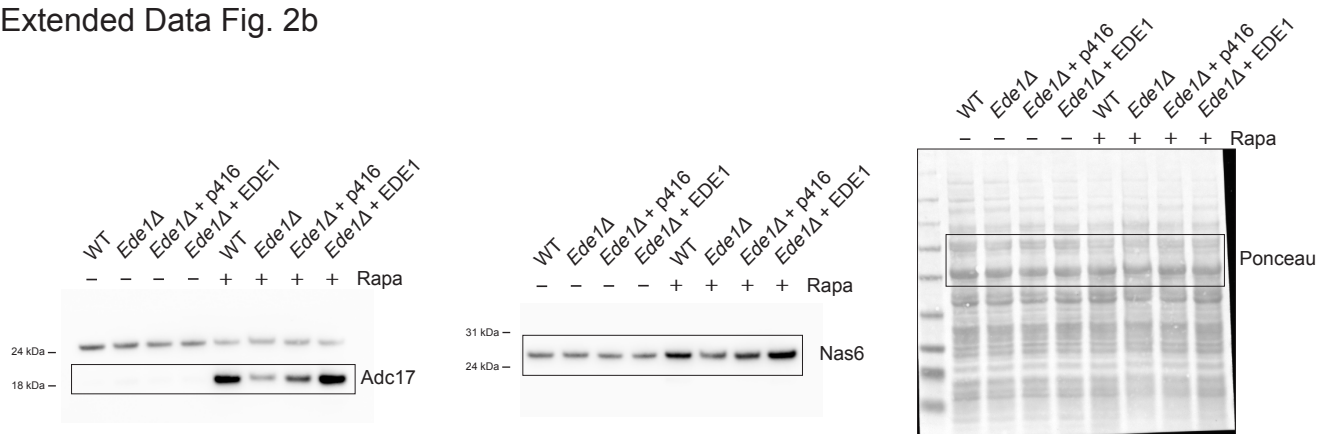

Extended Data Fig. 2c

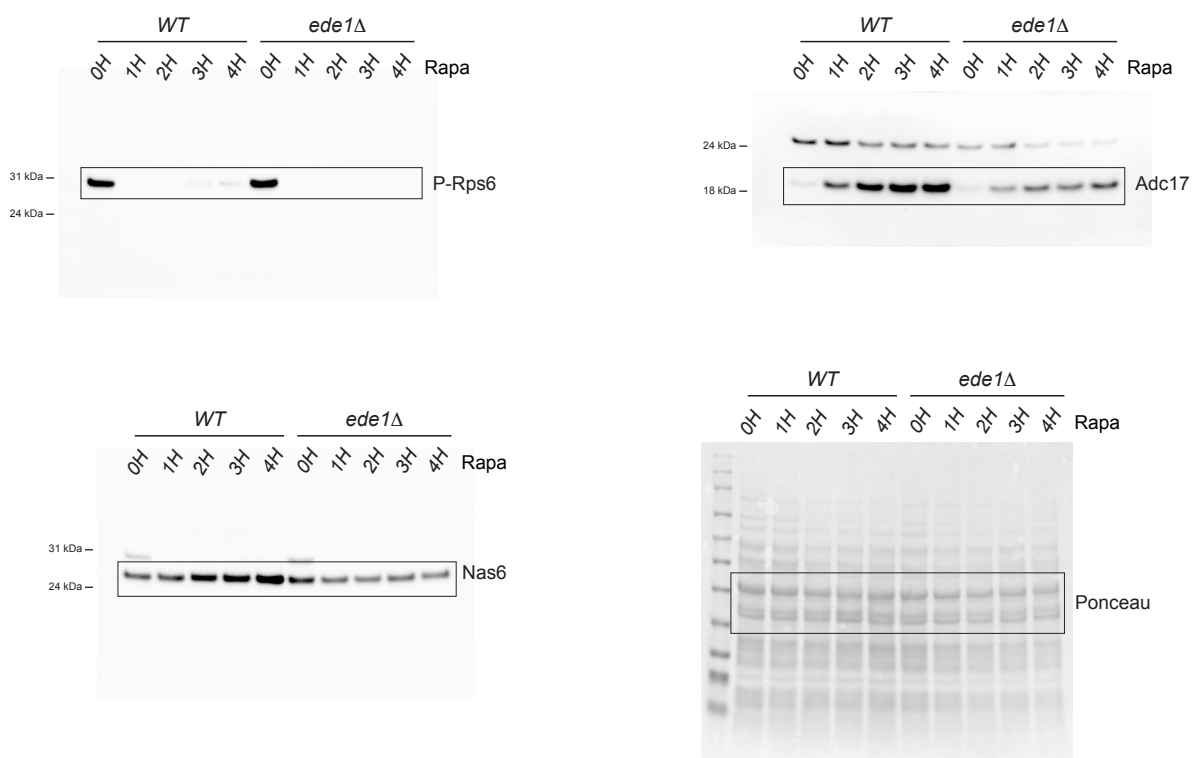

Supplement: Supplementary file 26 — Unprocessed western blots and/or gels. [file 41556_2022_938_MOESM26_ESM.pdf]

Extended Data Fig. 4b

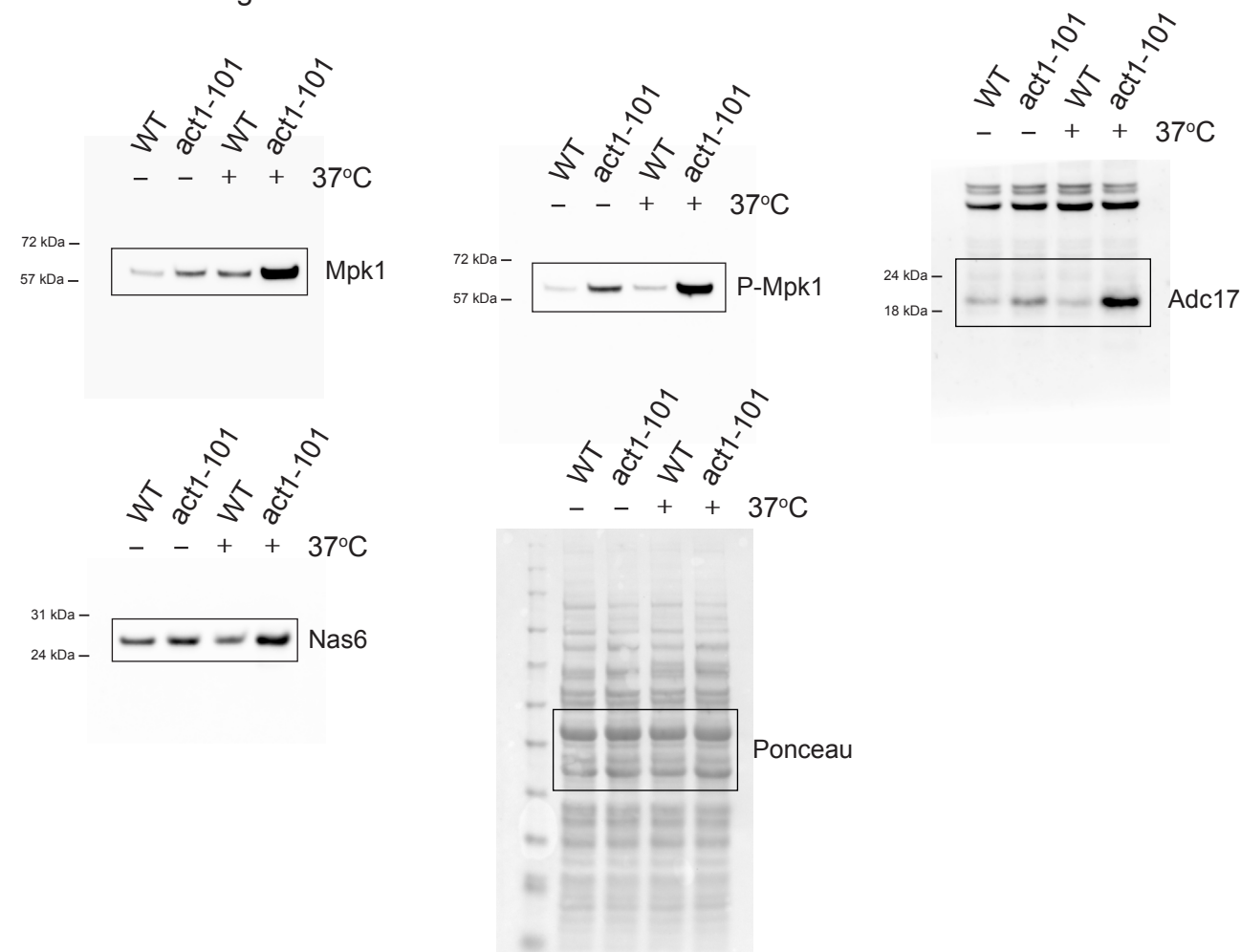

Extended Data Fig. 4e

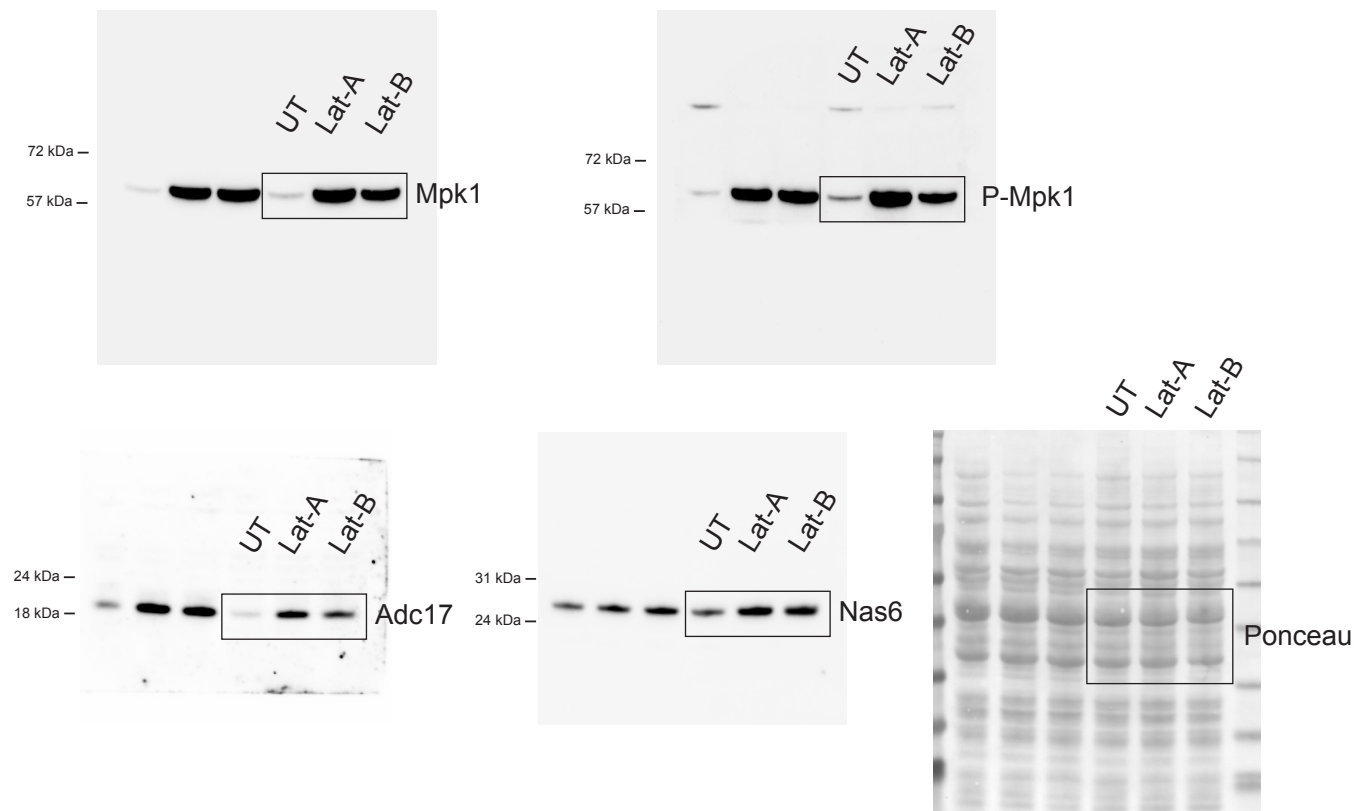

Supplement: Supplementary file 29 — Unprocessed western blots and/or gels. [file 41556_2022_938_MOESM29_ESM.pdf]
